# Alcohol induces TGFβ1 via downregulation of miR-1946a in murine lung fibroblast

**DOI:** 10.1038/s41598-020-76148-5

**Published:** 2020-11-05

**Authors:** Xian Fan, Stephen T. Mills, Mevelyn J. Kaalla, Viranuj Sueblinvong

**Affiliations:** 1 Department of Medicine, Division of Pulmonary, Allergy, Critical Care, and Sleep Medicine, Emory University School of Medicine, 615 Michael Street, Suite 205, Atlanta, GA 30322 USA; 2 Mercer University Health Sciences Center, Macon, GA 31207 USA

**Keywords:** Cell biology, Molecular biology

## Abstract

Exaggerated transforming growth factor-beta 1 (TGFβ1) expression worsens fibroproliferation following bleomycin-induced lung injury in alcohol-fed mice. MicroRNA (miR)-1946a is predicted to bind to the TGFβ1 3′ untranslated region (UTR), thereby inhibiting its transcription. We hypothesize that alcohol suppresses miR-1946a and induces TGFβ1. Primary murine lung fibroblasts (PLFs) were cultured ± alcohol, miR-1946a mimic or inhibitor, and TGFβ1 signaling inhibitors. miR-1946a was analyzed after alcohol treatment in vitro and in vivo. TGFβ1 expression and TGFβ1 3′UTR-luciferase activity was quantified. We showed that alcohol suppressed miR-1946a in the alcohol-fed mouse lungs and PLFs. MiR-1946a inhibitor increased TGFβ1 expression in the fibroblast. MiR-1946a mimic treatment suppressed TGFβ1 gene expression and TGFβ1 3′UTR activity. Overexpression of miR1946a inhibited alcohol-induced TGFβ1 gene and protein expression as well as alcohol-induced TGFβ1 and α-smooth muscle actin (SMA) protein expression in PLFs. In conclusion, miR-1946a modulates TGFβ1 expression through direct interaction with TGFβ1 3′UTR. These findings identify a novel mechanism by which alcohol induces TGFβ1 in the lung.

## Introduction

Tissue repair following an injury is a complex process that allows for replacement of dead or damaged cells thereby restoring normal tissue homeostasis. The repair process can be divided into three stages: an initial response, recovery, and resolution^1^. Multiple cell types, extracellular matrices, and cytokines are involved and tightly regulated. Under normal circumstances, the tissue repair process includes inflammatory cell migration to the injury site, expression of chemokines and cytokines, fibroblast migration to the injury site, generation of a provisional extracellular matrix (ECM), re-growth of epithelial cells, and subsequent apoptosis of activated fibroblasts^2^. However, when this tightly regulated process is disrupted, the result is unresolved inflammation leading to pathologic wound repair and subsequent organ dysfunction^3^. One of the critical factors in this overall balance is transforming growth factor beta-1 (TGFβ1) which is released in response to tissue injury, stimulates cell differentiation, and promotes wound healing^4^.

TGFβ1 has been shown to promote tissue fibrosis by inducing fibroblast-to-myofibroblast transdifferentiation and epithelial-to-mesenchymal transition (EMT) resulting in excessive collagen deposition and ultimately tissue fibrosis^5^. We previously showed that chronic alcohol ingestion promotes fibroproliferative changes and dysregulated repair following acute lung injury (ALI) that is associated with exaggerated constitutive and injury-provoked expression of TGFβ1^6^. Importantly, aberrant expression of TGFβ1 has been identified as a proximal event that mediates the ‘alcoholic lung’ phenotype, which includes redox imbalance, impaired epithelial barrier integrity, decreased alveolar macrophage immune function, and fibroproliferative disrepair following bleomycin-induced ALI^7–10^. However, the discrete mechanisms by which alcohol induces TGFβ1 expression remain poorly understood.

MicroRNAs (miRs) are small, non-coding RNAs that bind to the 3′ untranslated region (UTR), 5′ UTR, coding sequence (CDS), or promoter region of target messenger RNA (mRNA) sequences leading to mRNA degradation and decreased protein expression^11^. Although TGFβ1 expression is influenced by many different factors, several miRs have been shown to directly target TGFβ1^12–14^. Using in silico analysis, we identified that miR-1946a is predicted to bind directly to the 3′ UTR of TGFβ1 mRNA thereby inhibiting its transcription. Therefore, we hypothesize that alcohol induces TGFβ1 transcription by inhibiting miR-1946a expression.

## Methods

### Animals and chronic alcohol ingestion

Three-month old C57BL/6J wild type mice were obtained from Jackson Laboratories (Bar Harbor, ME). Mice were fed with or without alcohol for 8 weeks, then lungs were collected for miR analysis and primary lung fibroblasts (PLF) isolation as described below. All studies were approved by the Institutional Animal Care Use and Committee (IACUC) at Emory University and conformed to institutional and Association for Assessment and Accreditation of Laboratory Animal Care International (AAALAC) standards of medical sciences’ ethical principles and guidelines for the humane treatment of laboratory animals.

### Cell culture and treatment

Murine PLFs were isolated from the lungs of three-month old C57BL/6J wild type mice (Jackson Laboratories, Bar Harbor, ME) as previously described^15^. In short, lungs were removed and placed in the ice-cold sterile phosphate-buffered saline (PBS). The outer 3 mm of lung edge was removed, and the remaining lung was cut into approximately 1 mm cubes. Lung cubes were washed in ice-cold sterile PBS twice, after which they were transferred to the tissue culture dish while maintaining approximately 1 cm space between pieces. Complete culture media (Dulbecco's modified Eagle's medium (DMEM) (Cellgro, Manassas, VA), 4.5 g/L glucose supplemented with 20% fetal bovine serum (FBS), 100 U/ml penicillin, and 100 U/ml streptomycin) were added slowly to the dish without dislodging the pieces. They were incubated in 5% CO_2_ at 37 °C for 1–3 weeks to allow fibroblast migration out of tissue cubes. PLFs were used in experiment between 3 and 8 passages. Cells were subjected to several treatments, including alcohol (60 mM), TGFβ1 neutralizing antibody (2 µg/ml, R&D Systems, Minneapolis, MN), or activin receptor-like kinase 5 (ALK5 or TGFβ1 receptor type 1) inhibitor (SB431542, 8 µM, Sigma-Aldrich, St. Louis, MO) while in DMEM culture media supplement with 5% FBS and 100 U/ml penicillin/streptomycin.

### Overexpression and inhibition of miR-1946a

To assess whether miR-1946a mediates TGFβ1 expression, PLFs were transfected with synthetic miR-1946a mimic (5′AGCCGGGCAGUGGUGGCACACACUUUU; 5 nM, Qiagen, Valencia, CA), negative mimic (5 nM, Qiagen, Valencia, CA), anti-mmu-miR-1946a (miR-1946a inhibitor, 20 nM, Qiagen, Valencia, CA), or anti-miR negative control (20 nM, Qiagen, Valencia, CA) using Lipofectamine 3000 (Thermo Fisher Scientific, Waltham, MA) according to the manufacturer's protocol. Six hours after transfection, serum-free media was replaced with DMEM culture media supplemented with 5% FBS, and cells were incubated overnight. PLFs were treated with alcohol (60 mM) 24 h after transfection, and then continued incubation in culture media supplemented with 5% FBS for 24 or 72 h for gene and protein analysis (or 48 h after miR-1946a mimic or miR-1946a inhibitor treatment).

### Messenger RNA isolation and expression analysis

Messenger RNA was isolated from PLFs as previously described using a commercial RNA isolation kit according to the manufacturer’s protocol (Zymo Research, Irvine, CA)^16^. First-strand cDNA was synthesized, and quantitative PCR (qPCR) was performed with primers for 18 s and TGFβ1 using iQ SYBR Green Supermix (Bio-Rad, Hercules, CA). The iCycler (Bio-Rad, Hercules, CA) was utilized for real-time PCR analysis. The level of target mRNA expression was normalized to 18 s housekeeping gene levels and relative expression values were calculated as previously described^16^.

### MicroRNA isolation and expression analysis

The mirVana miR isolation kit was used to extract and purify microRNA (miR) according to the manufacturer’s protocol (Thermo Fisher Scientific, Waltham, MA). cDNA was synthesized from 250 ng of miR using the miScript II RT kit (Qiagen, Valencia, CA). qPCR was performed for SNORD95, miR-27a, miR-182, miR-378, miR-744, and miR-1946a expression with primers obtained from Qiagen using the QuantiTect SYBR Green PCR Kit. The level of target miR-27a, miR-182, miR-378, miR-744, and miR-1946a expression was normalized to SNORD95. The relative levels were determined by the comparative cycle threshold method as previously described^17^.

### Protein isolation and analysis

Total protein was isolated from PLF lysate as previously described^16^. Protein samples of equal amounts were separated on 4–15% gradient sodium dodecyl sulfate–polyacrylamide gel electrophoresis (SDS-PAGE) gel (Bio-Rad, Hercules, CA) and transferred to nitrocellulose membranes. The blots were exposed to blocking buffer (5% non-fat dry milk, 0.1% Tween 20 in Tris Buffered Saline (TBS), pH 7.4) for 1 h and then incubated with mouse anti-rabbit GAPDH (1:50,000, Sigma-Aldrich, St. Louis, MO), rat anti-mouse TGFβ1 (1:500, BD Pharmingen (BD Biosciences, San Jose, CA), or rabbit anti-alpha smooth muscle actin (α-SMA; 1:2,000, Abcam, Cambridge, MA) at 4 °C overnight. An appropriate horseradish peroxidase–conjugated secondary antibody (1:1,000, Amersham Biosciences, Pittsburgh, PA) was added prior to visualizing via enzyme-linked chemiluminescence using the SuperSignal West Pico kit (Pierce Biotechnology, Rockford, IL). Representative blots were cropped from full-length blots and combined for presentation using Photoshop CS5.

### TGFβ1 3′ UTR luciferase activity analysis

A total of 50,000 cells/well of PLFs were seeded in 96-well plates. PLFs were co-transfected with either luciferase reporter plasmid (pEZX-MT05) containing TGFβ1 3′UTR/Renilla luciferase plasmid or control plasmid/Renilla luciferase plasmid (Genecopoeia, Rockville, MD) using Lipofectamine 3000 (Thermo Fisher Scientific, Waltham, MA) according to the manufacturer's protocol. Twenty-four hours after plasmid transfection, cells were transfected with synthetic miR-1946a mimic (5 nM) or negative mimic (5 nM) from Qiagen as outlined above. Forty-eight hours later, a dual-luciferase reporter assay was performed (Promega Corp., Madison, WI), TGFβ1 3′ UTR luciferase activity values were expressed as ratios of arbitrary units of firefly luciferase/renilla luciferase activity.

### Statistical analyses

Unpaired two tailed t-tests or one-way ANOVA was utilized for comparisons between groups using GraphPad Prism and GraphPad InStat version 7 (San Diego, CA). If statistical significance was reached following one-way ANOVA, post-test analysis using Dunnett’s correction method was performed. GraphPad Prism and GraphPad InStat version 7 were used to calculate statistics. Significant differences were accepted at a *P* value of < 0.05.

### Ethics approval

All studies were approved by the IACUC at Emory University and conformed to institutional standards and the Swiss Academy of Medical Sciences’ ethical principles and guidelines for the humane treatment of laboratory animals.

## Results

### Chronic alcohol ingestion attenuates miR-1946a in the murine lung

**Figure 1:**
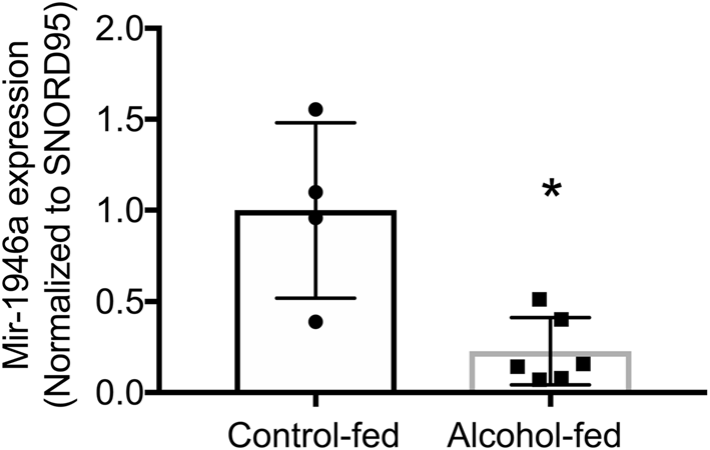
Chronic alcohol ingestion attenuates miR-1946a in the murine lung. Alcohol‐fed mice were sacrificed after 8 weeks of alcohol ingestion along with their control-fed counterparts. The lungs were harvested and analyzed for miR-1946a gene expression by quantitative PCR. N = 4–6. *P < 0.05 compared to lungs from control-fed mice.

We previously showed that chronic alcohol ingestion exaggerated TGFβ1 expression through an augmentation of miR-21 expression, in both the lung and lung fibroblasts^6,17^. However, inhibition of miR-21 resulted in partial suppression of TGFβ1^17^. We performed analysis in silico with Target Scan and miRWalk to identify miRs which could interact with TGFβ1 3′UTR (NM_000660). Several miRs were identified including miR-1946a which has two potential binding sites on conserved sites of TGFβ1 3′UTR. To evaluate the effect of alcohol ingestion on miR-1946a expression, we determined its expression in the lungs of control-fed versus alcohol-fed mice. There is ~ 60% decrease in miR-1946a expression in chronic alcohol-fed mice lungs when compared to control-fed mice lungs (*P* < 0.05, Fig. 1). This data suggest that chronic alcohol ingestion suppressed overall miR-1946a expression in the murine lung.

### Alcohol exposure attenuates miR-1946a expression in murine primary lung fibroblasts

**Figure 2:**
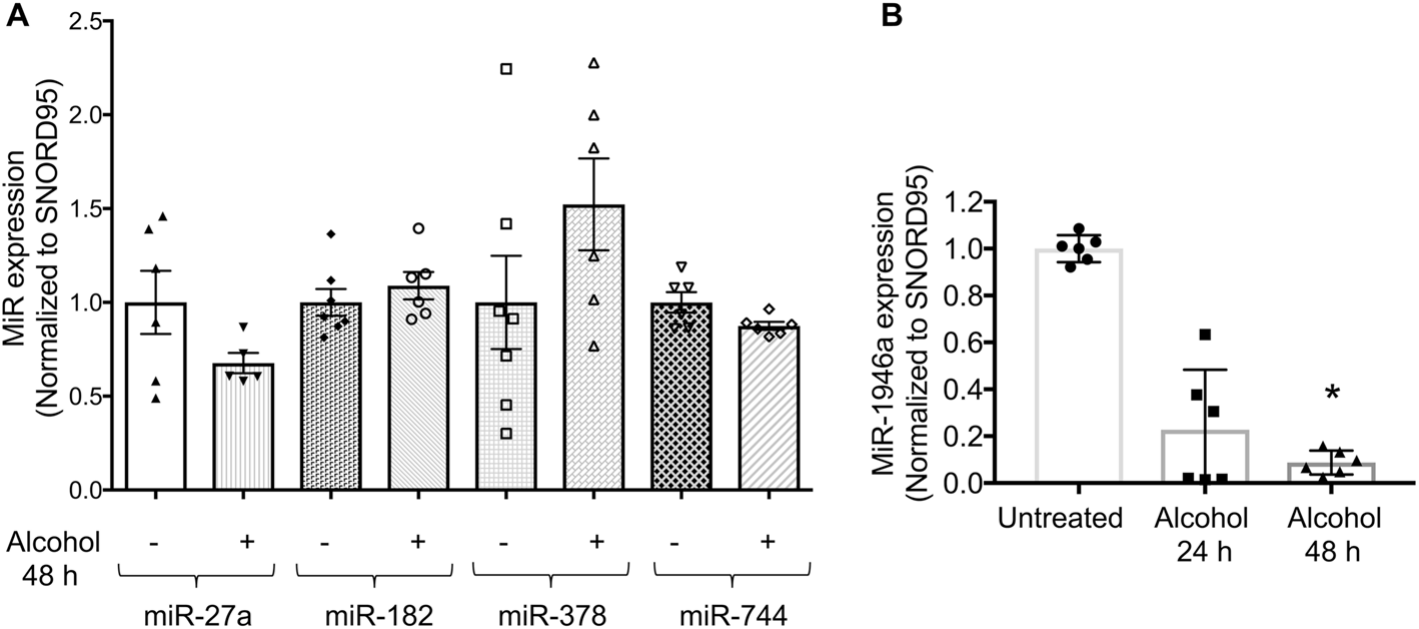
Alcohol exposure attenuates miR-1946a expression in murine primary lung fibroblasts. Mouse primary lung fibroblasts (PLFs) were isolated from C57BL6/J wild-type mice. PLFs (between passage 3 and 8) were treated with or without alcohol (60 mM). (**A**) At 48 h, cells were harvested for miR-27a, miR-182, miR-378, and miR-744 expression analysis by quantitative PCR. (**B**) At 24 and 48 h, cells were harvested for miR-1946a gene expression analysis by quantitative PCR. N = 6. *P < 0.05, increased compared to untreated cells.

The lung is comprised of several cell types. Lung fibroblasts are the main producers and regulators of the TGFβ1. To explore the effect of alcohol on TGFβ1 modulating miRs expression in murine PLFs, we assessed the expression of several miRs including miR-21^17^, miR-27a^18^, miR-182^19^, miR-378^20,21^, miR-744^14^, and miR-1946a. We found that alcohol increased miR-21 expression^17^ but did not alter the expression of miR-27a, miR-182, miR-378, and miR-744 (Fig. 2A). However, alcohol treatment led to an approximate 90% decrease in miR-1946a expression after 48 h of alcohol treatment in PLFs (*P* < 0.05, Fig. 2B). These results indicate that alcohol treatment suppresses miR-1946a expression in PLFs.

### MiR-1946a mediates TGFβ1 expression in murine primary lung fibroblasts

**Figure 3:**
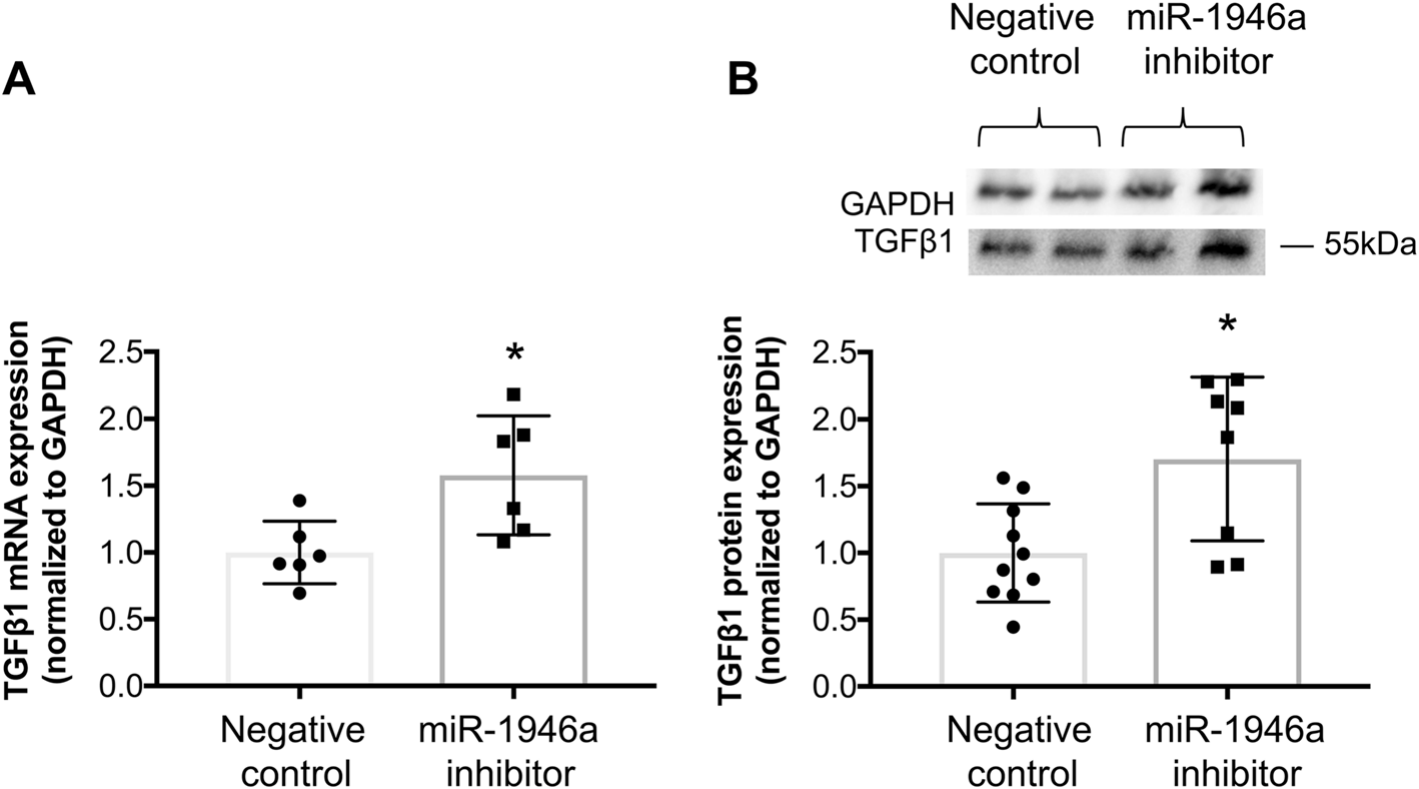
Inhibition of miR-1946a induces TGFβ1 gene and protein expression in murine primary lung fibroblasts. Mouse PLFs were isolated from C57BL6/J wild-type mice, and cells (between passage 3 and 8) were transfected with anti-mmu-miR-1946a (miR-1946a inhibitor) or inhibitor negative control (20 nM) using lipofectamine 3000. (**A**) At 48 h following a transfection, cells were collected for TGFβ1 gene expression analysis by quantitative PCR (N = 6), and (**B**) at 72 h following a transfection, cells were collected for TGFβ1 protein expression analysis by Western Immunoblot (N = 8–10). Representative gels shown above summary data. *P < 0.05 compared to cells transfected with negative controls.

**Figure 4:**
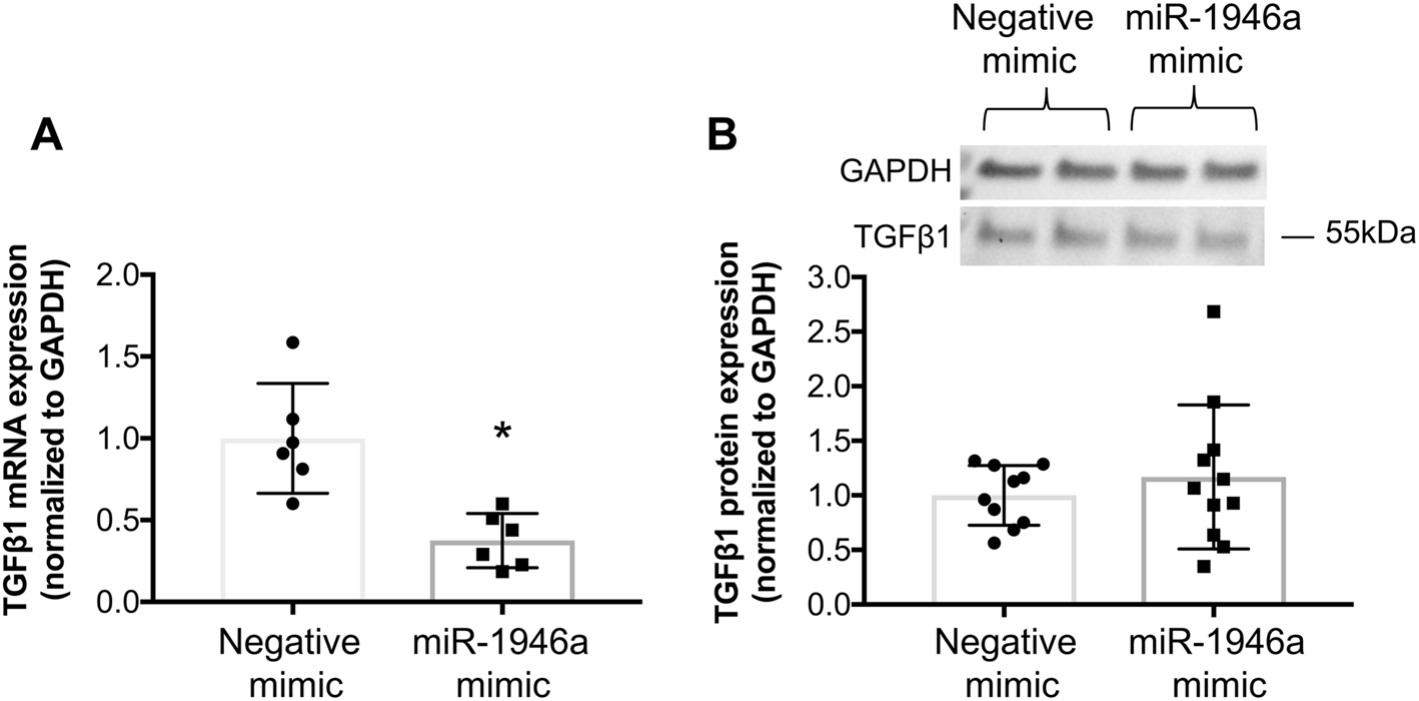
MiR-1946a overexpression suppresses TGFβ1 gene expression but not protein expression in murine primary lung fibroblasts. Mouse PLFs were isolated from C57BL6/J wild-type mice, and cells (between passage 3 and 8) were transfected with synthetic miR-1946a mimics or negative mimic (5 nM) using lipofectamine 3000. (**A**) At 48 h following transfection, cells were collected for TGFβ1 gene expression analysis by quantitative PCR (N = 6), and (**B**) at 72 h following a transfection, cells were collected for TGFβ1 protein expression analysis by Western Immunoblot (N = 11). Representative gels shown above summary data. *P < 0.05 compared to cells transfected with negative mimic.

To ascertain whether miR-1946a mediates TGFβ1 expression in PLFs, cells were transfected with synthetic miR-1946a mimic or miR-1946a inhibitor. We found that inhibiting miR-1946a with a miR-1946a inhibitor significantly up-regulated TGFβ1 gene (*P* < 0.05, Fig. 3A) and protein (*P* < 0.05, Fig. 3B). In parallel, overexpression of miR-1946a with miR-1946a mimic suppressed TGFβ1 gene expression (*P* < 0.05, Fig. 4A) but not TGFβ1 protein expression (Fig. 4B).

**Figure 5:**
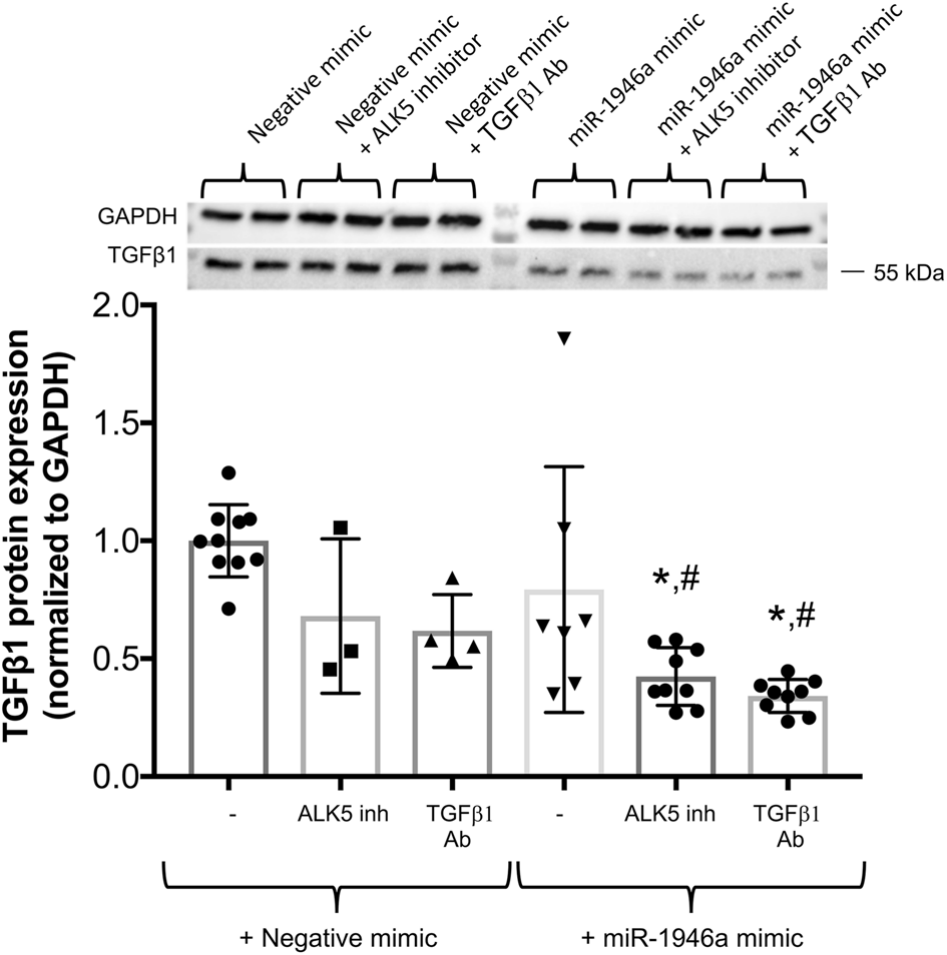
MiR-1946a overexpression attenuates TGFβ1 protein expression in the murine primary lung fibroblasts when the TGFβ1 signaling pathway is disrupted. Mouse PLFs (between passage 3 and 8) were treated with activin receptor-like kinase 5 (ALK5) inhibitors (SB431542, 8 µM) or TGFβ1 neutralizing antibody (TGFβ1 Ab) (2 µg/ml). Cells were incubated overnight then transfected with synthetic miR-1946a mimic (5 nM) or negative mimic (5 nM). At 72 h, total protein was isolated and analyzed for TGFβ1 protein expression. Representative gels shown above summary data. *P < 0.05 compared to cells transfected with negative mimic. ^#^*P* < 0.05 compared to cells treated with ALK5 inhibitors or TGFβ1 Ab + negative mimic. N = 3–10.

Several factors can influence TGFβ1 protein expression and activation including an autocrine mechanism and activation of a non-canonical pathway resulting in an increase in TGFβ1 protein expression independent of its gene transcription^22,23^. We hypothesized that activation of baseline expression of TGFβ1 lead to a perpetuation cycle of protein translation despite of an attenuation of TGFβ1 mRNA by miR-1946a mimic. To test this hypothesis, we treated cells with TGFβ1 receptor 1 inhibitor (activin receptor-like kinase 5 (ALK5) inhibitors) or TGFβ1 neutralizing antibody prior to miR-1946a overexpression. In Fig. 5, we showed that treatment of cells with TGFβ1 signaling inhibitors or miR-1946a overexpression individually did not result in any significant changes in TGFβ1 protein expression. However, a combination of miR-1946a mimics and TGFβ1 signaling inhibitors significantly attenuated TGFβ1 protein expression when compared to negative controls (*P* < 0.05, Fig. 5). This data indicates that autocrine TGFβ1 partially contributed to the baseline expression of TGFβ1.

### MiR-1946a decreased TGFβ1 activity by interacting with the TGFβ1 3′UTR

**Figure 6:**
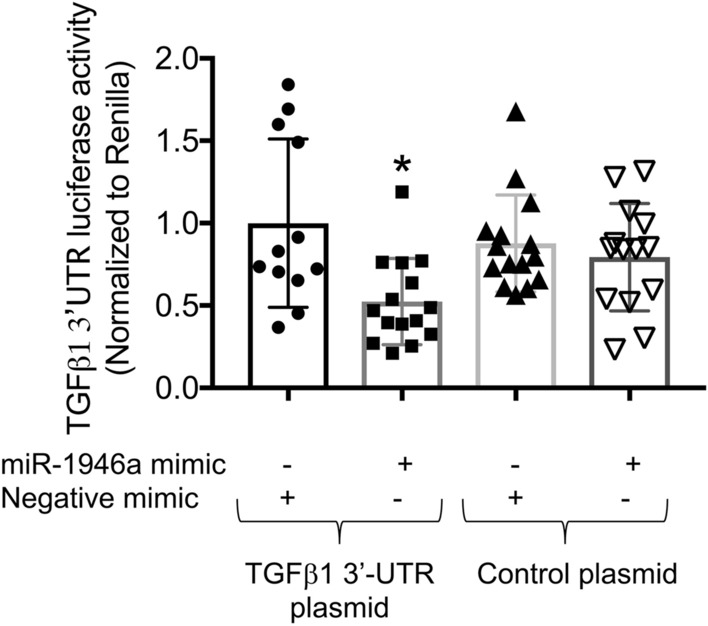
Overexpression of miR-1946a inhibits TGFβ1 3′UTR luciferase activity. Mouse PLFs were isolated from C57BL6/J wild-type mice, PLFs (between passage 3 and 8) were co-transfecting PLFs with the firefly luciferase-expressing plasmid (pEZX-MT05) containing wild-type TGFβ1 3′UTR and miR-1946a mimic (or appropriate negative control plasmid and negative mimic) using Lipofectamine 3000. Cells were incubated for 24 h and then transfected with synthetic miR-1946a mimics or negative mimic. Forty-eight hours after transfection, TGFβ1 3′UTR luciferase activity relative to Renilla luciferase activity was quantified. N = 12–15. *P < 0.05 compared to cells transfected with wild-type TGFβ1 3′UTR plasmid + negative mimic controls.

We next sought to determine whether or not miR-1946a mediates TGFβ1 via direct interaction with TGFβ1 3′UTR. By co-transfecting PLFs with the firefly luciferase-expressing plasmid (pEZX-MT05) containing wild-type TGFβ1 3′UTR and miR-1946a mimic (or appropriate negative control plasmid and negative mimic), we demonstrated that miR-1946a mimic significantly suppressed luciferase activity in cells transfected with plasmid containing wild-type TGFβ1 3′UTR (*P* < 0.05). MiR-1946a mimic had no effect on cells transfected with control plasmid (Fig. 6). This data implies that miR-1946a attenuates TGFβ1 expression via a direct interaction with TGFβ1 3′UTR.

### Overexpression of miR-1946a inhibits alcohol-induced TGFβ1 and α-smooth muscle actin (α-SMA) expression in murine lung fibroblasts

**Figure 7:**
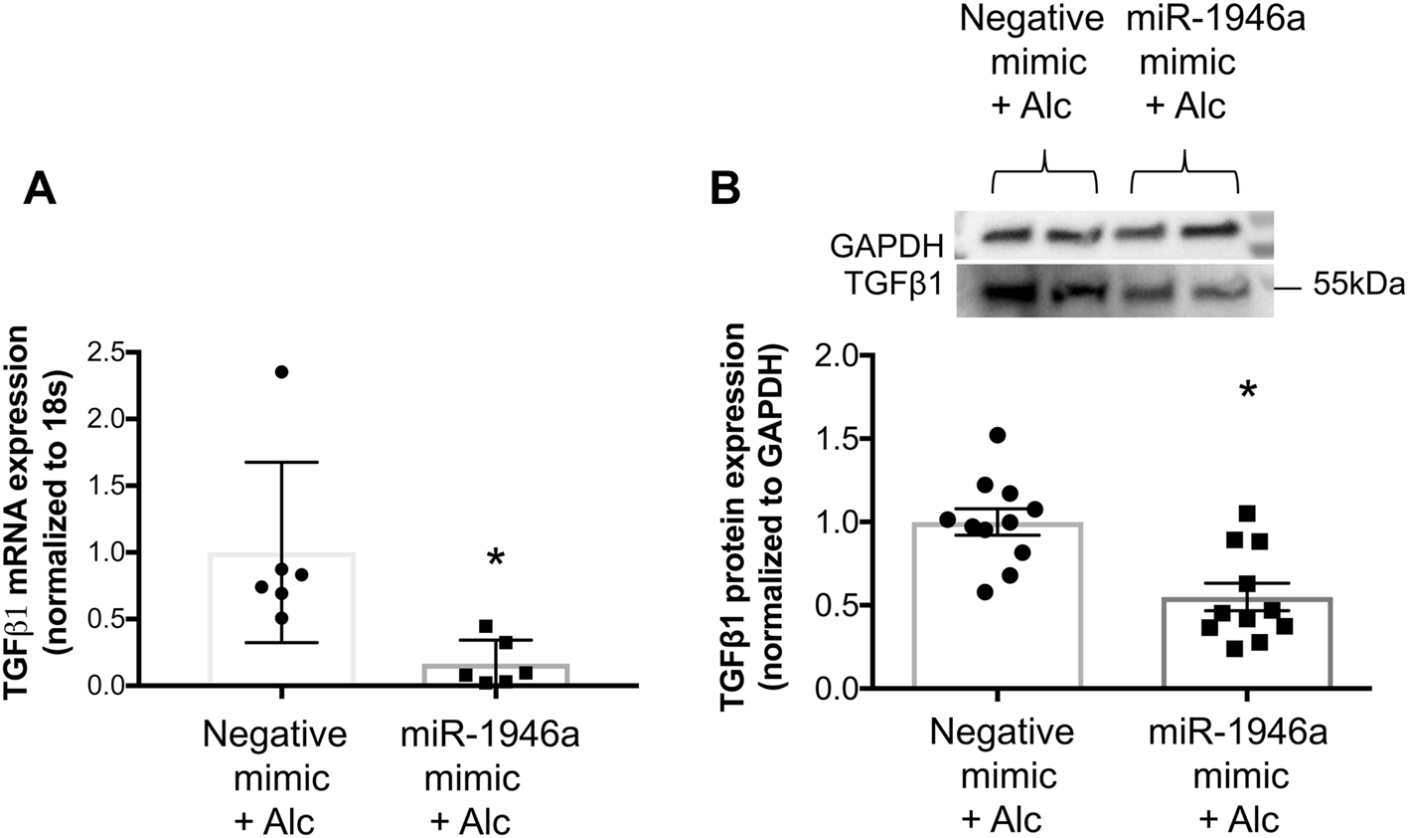
MiR-1946a overexpression attenuates alcohol-induced TGFβ1 gene and protein expression in murine primary lung fibroblasts. Mouse PLFs (between passage 3 and 8) were transfected with synthetic miR-1946a mimic or negative mimic (5 nM) using Lipofectamine 3000. Following a 24-h incubation, cells were exposed to alcohol (60 mM). (**A**) at 24 h following alcohol exposure, cells were collected for TGFβ1 gene expression analysis by qPCR (N = 6), and (**B**) at 72 h following alcohol exposure, cells were collected for TGFβ1 protein expression analysis by Western Immunoblot (N = 11). Representative gels shown above summary data. *P < 0.05 compared to cells transfected with negative mimic and exposed to alcohol.

**Figure 8:**
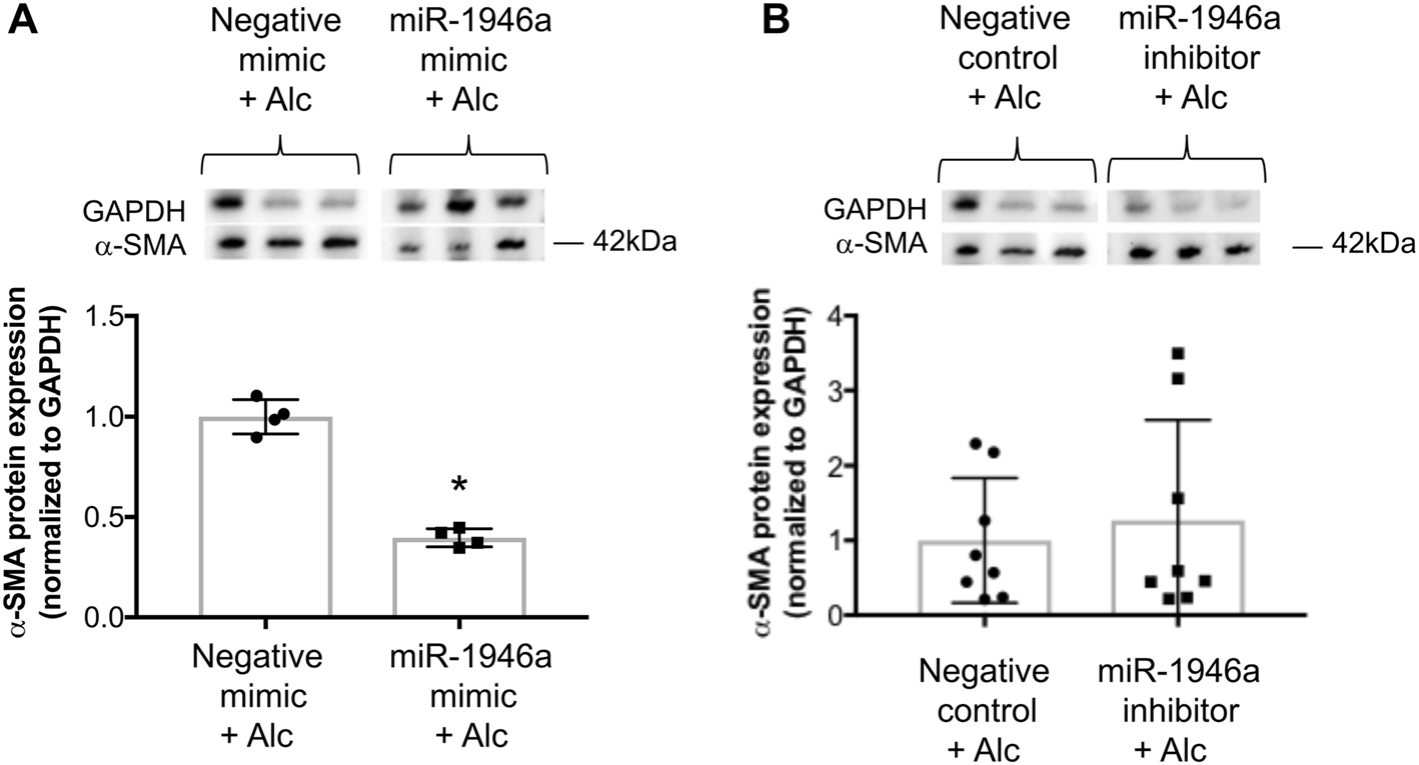
MiR-1946a overexpression attenuates alcohol-induced alpha-smooth muscle actin (α-SMA) expression in murine primary lung fibroblasts. Mouse PLFs (between passage 3 and 8) were transfected with (**A**) synthetic miR-1946a mimic (5 nM) or negative mimic (5 nM), (**B**) anti-mmu-miR-1946a (miR-1946a inhibitor, 20 nM) or negative controls (20 nM) using Lipofectamine 3000. Following a 24-h incubation, cells were exposed to alcohol (60 mM), and at 72 h following alcohol exposure, cells were collected for α-SMA protein expression analysis by Western Immunoblot. Representative gels shown above summary data. *P < 0.05 compared to cells transfected with negative mimic and exposed to alcohol. N = 4–8.

To assess whether overexpression of miR-1946a could attenuate alcohol-induced TGFβ1 in PLFs, cells were transfected with the miR-1946a mimic and treated with alcohol (60 mM). Cells were collected for gene and protein expression analyses at 24 or 72 h following treatment, respectively. We showed that miR-1946a overexpression inhibited alcohol-induced TGFβ1 gene and protein expression (*P* < 0.05, Fig. 7). To evaluate the effect of miR-1946a on TGFβ1 function, cells were transfected with miR-1946a mimic or inhibitor transfection, treated with alcohol, and then analyzed for α-SMA protein expression. In Fig. 8, we showed that miR-1946a overexpression attenuated alcohol-induced α-SMA protein expression (*P* < 0.05, Fig. 8A). However, inhibition of miR-1946a did not augment the effect of alcohol on α-SMA protein expression (Fig. 8B).

## Discussion

Chronic alcohol ingestion causes a marked increase of TGFβ1 expression in rodent lung fibroblasts^6^. We speculated that one of the mechanisms by which alcohol induces TGFβ1 expression is through modulation of miRs. In this study, we found that miR-1946a gene expression was suppressed in the lungs of alcohol-fed mice and alcohol-exposed murine PLFs. When the miR1946a mimic and inhibitor were introduced ex vivo in PLF, it showed an inverse relationship between miR-1946a and TGFβ1 expression. MiR1946a inhibitor significantly increased the TGFβ1 gene and protein expression in the PLF. In parallel, overexpression of miR1946a inhibited TGFβ1 gene expression. Furthermore, overexpression of miR-1946a attenuated TGFβ1 3′ untranslated region (UTR) luciferase activity in PLFs. More importantly, overexpression of miR-1946a prevented alcohol-induced TGFβ1 and its downstream effector, α-SMA expression in the murine PLFs. Taken together, these results demonstrate that one of the mechanisms by which alcohol induces TGFβ1 expression is through attenuation of miR-1946a expression. The mechanism by which miR-1946a regulates TGFβ1 expression is likely through direct interaction with the 3′UTR of the TGFβ1 gene.

TGFβ1 promotes tissue fibrosis by inducing fibroblast-myofibroblast transdifferentiation, thereby resulting in excessive collagen deposition and, ultimately, tissue fibrosis^5,24–27^. We showed that chronic alcohol ingestion primes the lung for fibroproliferative disrepair following bleomycin-induced acute lung injury through an exaggerated expression of TGFβ1^6^. TGFβ1 expression and signaling are mediated by many miRs^28^. Several miRs modulate TGFβ1 signaling by either directly targeting TGFβ1 mRNA or its signaling pathway^28,29^. In our previous study, we showed that alcohol up-regulated miR-21. However, inhibition of miR-21 partially attenuated alcohol-induced TGFβ1 expression implying that there are other factors in the play^17^. Based on the publications^13,14,18,20,21,30^ and in silico analysis, we here analyzed several miRs in our experimental model. Interestingly, alcohol exposure attenuated miR-1946a in the whole lung and PLFs but did not alter miR-27a^18^, miR-182^19,30^, miR-378^20,21^, miR-744^14^ which were previously shown to modulate TGFβ1 in other experimental models. To our knowledge, this is the first report linking alcohol exposure with inhibition of miR-1946a in the lung and PLFs.

The complexity of the TGFβ1 cascade includes its ability to self-activate in an autocrine fashion and non-canonical pathway independent of gene transcription^22,31,32^. In this study, miR-1946a mimic resulted in an attenuation of TGFβ1 gene expression but did not affect TGFβ1 protein expression. We speculated that when miR-1946a suppressed TGFβ1 gene expression, it activated baseline TGFβ1 causing upregulation of non-canonical pathway leading to persistent TGFβ1 protein expression independent of TGFβ1 transcription^23^. To determine the interplay between TGFβ1 and miR-1946a independent of TGFβ1 self-activation, we inhibited the TGFβ1 signaling pathway before overexpressing miR-1946a. The data supported our speculation and showed that a combination of miR-1946a and TGFβ1 signaling pathway inhibitors led to significant suppression of TGFβ1 protein expression. These data highlighted the complexity of the TGFβ1 pathway, and it is essential to consider a combination of interventions to mitigate the function of TGFβ1.

MicroRNAs (miRs) are a class of non-coding RNAs that were shown to regulate gene expression at the post-transcriptional level^33^. They regulate gene expression by binding to specific regions of the targeted mRNA, including the 3′ untranslated region (3′UTR), the 5′ UTR, coding sequences, and the promoter region^34^. MiR-1946a is predicted to have two binding sites on TGFβ1 3′UTR, and possible binding sites on TGFβ1 5′UTR and CDS region. By using TGFβ1 3′UTR luciferase assays, we showed that miR-1946a can suppress TGFβ1 mRNA by directly interact with its 3′UTR. Although this study confirmed that miR-1946a interact with TGFβ1 3′UTR, it does not rule out other binding sites i.e. 5′UTR or CDS region. A future study to further elucidate miR-1946a and TGFβ1 interaction will be needed.

Taken together, we present new evidence that alcohol suppresses miR-1946a in the mouse lung and PLFs through direct interaction with TGFβ1 3′UTR. Our findings provide new insight into the mechanism by which alcohol amplifies TGFβ1 expression in the lung. There are still several unanswered questions, including the mechanisms by which alcohol modulate miRs expression, the optimal balance, and interaction between several TGFβ1 regulators, and what are the other miR-1946a targets that could affect TGFβ1 signaling. Future studies are warranted to elucidate the intricate pathways involved in TGFβ1 regulation by alcohol with the goal of developing effective therapeutic options in the at-risk population.

## Supplementary information

Supplementary Information.

## Data Availability

All data and materials are in compliance with the scientific standards. No datasets were generated or analyzed during the current study.
