# Supplementary material for: Alcohol induces TGFβ1 via downregulation of miR-1946a in murine lung fibroblast
Source: Sci Rep. 2020 Nov 5;10:19089. doi: 10.1038/s41598-020-76148-5 (PMC7644620; doi:10.1038/s41598-020-76148-5)

# Supplementary data for Alcohol induces TGFβ1 via downregulation of miR-1946a in murine lung fibroblast

Running Title: Alcohol induces TGFβ1 via miR-1946a

Xian Fan, MD[1], Stephen T Mills, BS[1], Mevelyn J Kaalla, BS[2],

and Viranuj Sueblinvong, MD[1]

[1]Emory University School of Medicine, Department of Medicine, Division of Pulmonary, Allergy, Critical Care, and Sleep Medicine

Atlanta, GA 30322

[2]Mercer University School of Medicine, Macon, GA 31207

**Corresponding author**: Viranuj Sueblinvong, MD (ORCID ID 000-0002-4770-5900)

615 Michael Street, Suite 205

Atlanta, GA 30322

Email: vsuebli@emory.edu

## SUPPLEMENTARY FIGURE LEGENDS

**Supplementary data for Figure 3. Inhibition of miR-1946a induces TGFβ1 gene and protein expression in murine primary lung fibroblasts.** Mouse PLFs were isolated from C57BL6/J wild-type mice and cells (between passage 3-8) were transfected with anti-mmu-miR-1946a (miR-1946a inhibitor) or inhibitor negative control (20 nM) using lipofectamine 3000. At 72 hours following a transfection, cells were collected for TGFβ1 and GAPDH protein expression analysis by Western Immunoblot (N = 8-10). Representative full-length gels are shown.

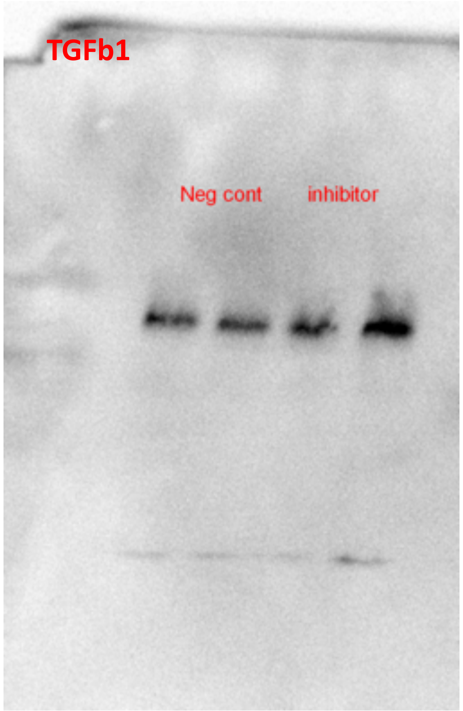


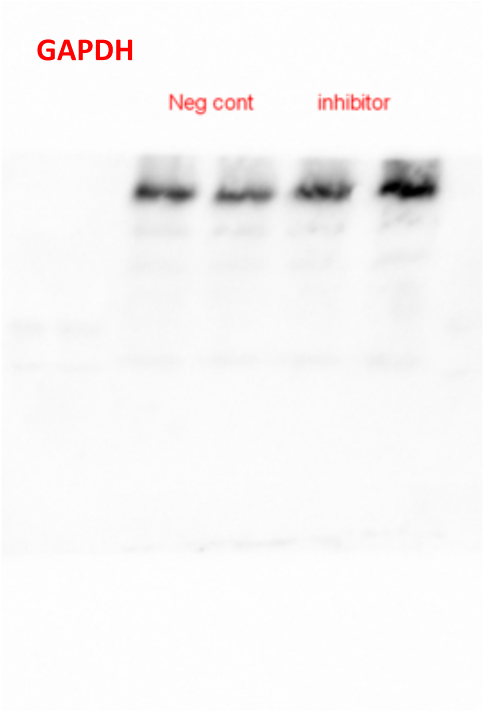

**Supplementary data for Figure 4. MiR-1946a overexpression suppresses TGFβ1 gene expression but not protein expression in murine primary lung fibroblasts.** Mouse PLFs were isolated from C57BL6/J wild-type mice and cells (between passage 3-8) were transfected with synthetic miR-1946a mimics or negative mimic (5 nM) using lipofectamine 3000. At 72 hours following a transfection, cells were collected for TGFβ1 and GAPDH protein expression analysis by Western Immunoblot (N = 11). Representative full-length gels are shown.

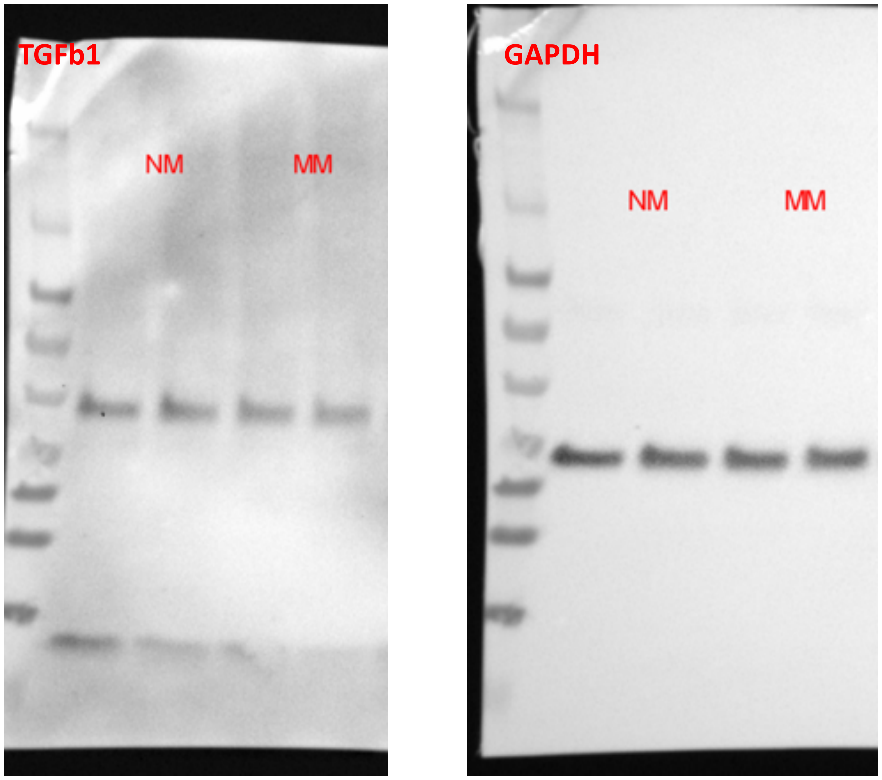

**Supplementary data for Figure 5. MiR-1946a overexpression attenuates TGFβ1 protein expression in the murine primary lung fibroblasts when the TGFβ1 signaling pathway is disrupted.** Mouse PLFs (between passage 3-8) were treated one time with activin receptor-like kinase 5 (ALK5) inhibitors (SB431542, 8 μM) or TGFβ1 neutralizing antibody (2 μg/ml). Cells were incubated overnight then transfected with synthetic miR-1946a mimic (5 nM) or negative mimic (5 nM). At 72 hours, total protein was isolated and analyzed for TGFβ1 and GAPDH protein expression. Representative full-length gels are shown.

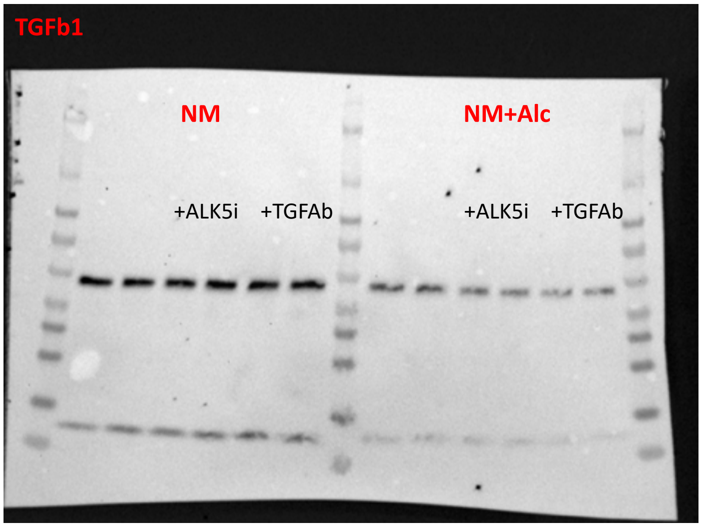


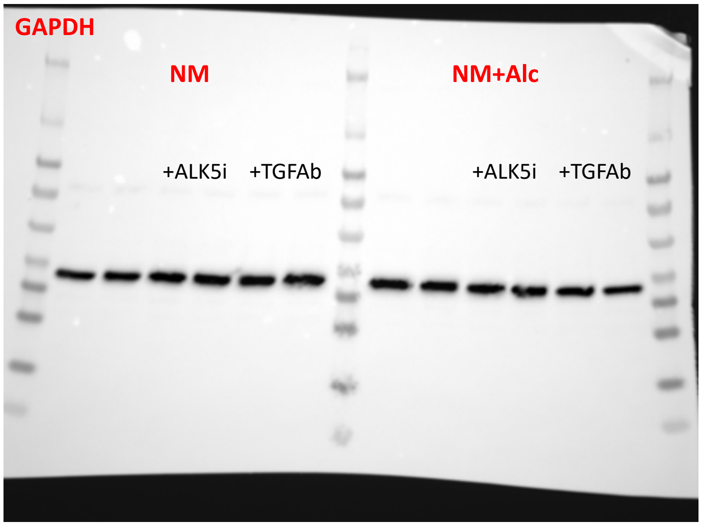

**Supplementary data for Figure 7. MiR-1946a overexpression attenuates alcohol-induced TGFβ1 gene and protein expression in murine primary lung fibroblasts.** Mouse PLFs (between passage 3-8) were transfected with synthetic miR-1946a mimic or negative mimic (5 nM) using Lipofectamine 3000. Following a 24-hour incubation, cells were exposed to alcohol (60 mM). At 72 hours following alcohol exposure, cells were collected for TGFβ1 and GAPDH protein expression analysis by Western Immunoblot (N = 11). Representative full-length gels are shown.

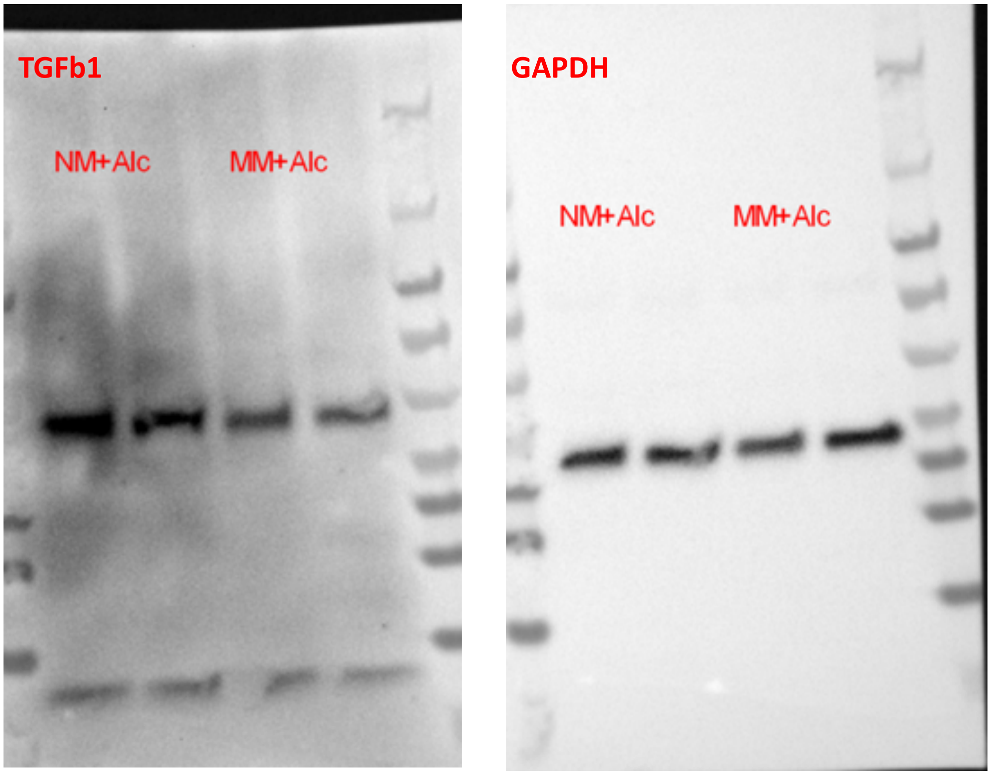

**Supplementary data for Figure 8. MiR-1946a overexpression attenuates alcohol-induced alpha-smooth muscle actin ($\alpha$-SMA) in murine primary lung fibroblasts.** Mouse PLFs (between passage 3-8) were transfected with (A) synthetic miR-1946a mimic (5 nM) or negative mimic (5 nM), (B) anti-mmu-miR-1946a (miR-1946a inhibitor, 20 nM) or negative controls (20 nM) using Lipofectamine 3000. Following a 24-hour incubation, cells were exposed to alcohol (60 mM) and at 72 hours following alcohol exposure, cells were collected for $\alpha$-SMA and GAPDH protein expression analysis by Western Immunoblot. Representative full-length gels are shown and red boxes highlighted the group used in data analysis.

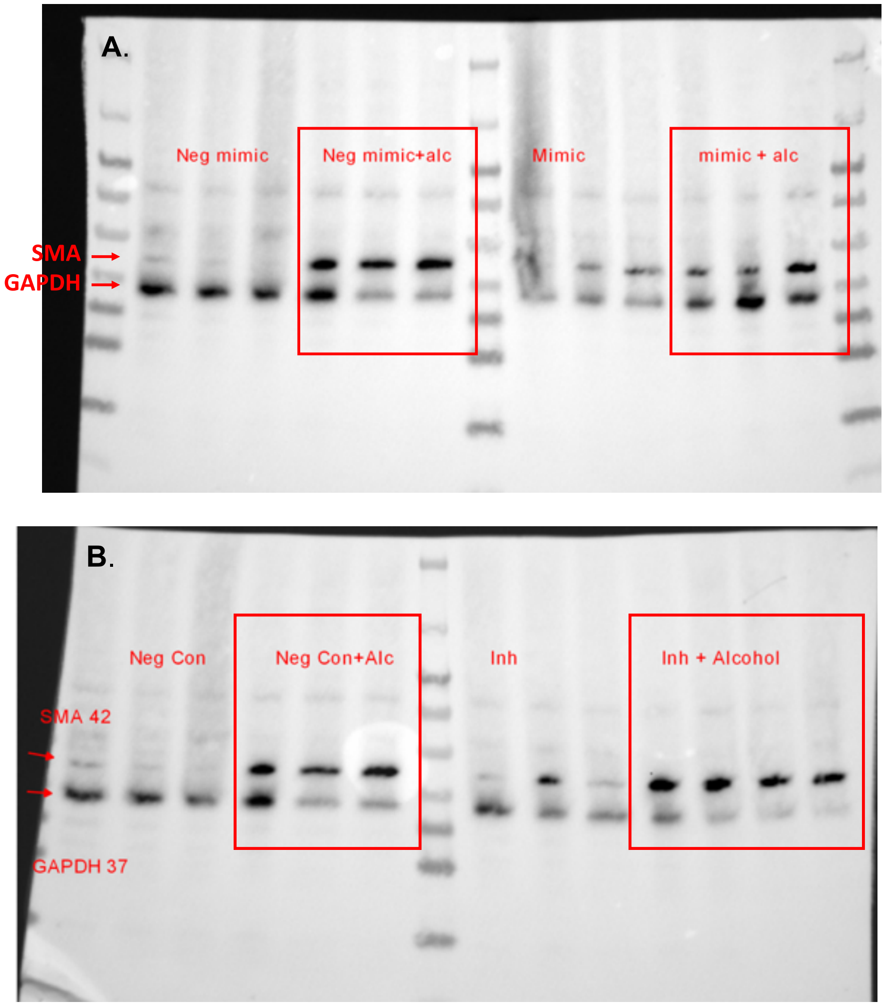

Supplement: Supplementary file 1 — Supplementary Information. [file 41598_2020_76148_MOESM1_ESM.pdf]
